# Evaluating Employment Quality as a Determinant of Health in a Changing Labor Market

**DOI:** 10.7758/RSF.2019.5.4.09

**Published:** 2019-09

**Authors:** TREVOR PECKHAM, KAORI FUJISHIRO, ANJUM HAJAT, BRIAN P. FLAHERTY, NOAH SEIXAS

**Affiliations:** environmental and occupational health sciences and clinical instructor in health services at the University of Washington.; epidemiologist at the National Institute for Occupational Safety and Health.; epidemiology at the University of Washington.; psychology at the University of Washington.; environmental and occupational health sciences at the University of Washington.

**Keywords:** employment quality, occupational health, latent class analysis, mental health, work-related injury

## Abstract

The shifting nature of employment in recent decades has not been adequately examined from a public health perspective. To that end, traditional models of work and health research need to be expanded to include the relational and contractual aspects of employment that also affect health. We examine the association of three health outcomes with different types of employment in the contemporary U.S. labor market, as measured by a multidimensional construct of employment quality (EQ) derived from latent class analysis. We find that EQ is associated with self-rated health, mental health, and occupational injury. Further, we explore three proposed mediating mechanisms of the EQ-health relationship (material deprivation, employment-related stressors, and occupational risk factors), and find each to be supported by these data.

Rapid technological innovation, globalization processes, economic recessions, and demographic changes over the past several decades have caused a number of adaptive changes in the labor market, including the fundamental transformation of the nature and organization of work (Bosch 2004; Kalleberg 2009). Most no-table is the shift away from maintaining a stable workforce toward more flexible and economically competitive employment practices (Benach et al. 2014; Bosch 2004; Kalleberg 2009; Weil 2014). Consequently, the number of workers in permanent, full-time, regularly scheduled work with secure wages and benefits has declined; and concurrently, nonstandard arrangements have increased (Howard 2016; Kalleberg 2000). In addition to the growth of atypical forms of employment, other dimensions of work also became destandardized, including working hours, opportunities for advancement, and worker-employer relations (Scott-Marshall and Tompa 2011). These changes have far-reaching consequences for the labor market experiences of millions of Americans; however, they have not been adequately examined from a public health perspective and compel the need for a new understanding of the elements of jobs that contribute to poor health (Peckham et al. 2017; Scott-Marshall and Tompa 2011; Tompa et al. 2007). This paucity of research reflects the typical exclusion of occupation as a primary social determinant of health (Ahonen et al. 2018), as well as lack of measures that adequately capture employment conditions. This study offers an initial exploration of health consequences of different types of employment in the contemporary U.S. labor market as measured by a multidimensional construct of employment quality. Further, we explore potential mechanisms by which EQ affects health.

We begin by clarifying important terms for interdisciplinary audiences. Although often used interchangeably, *job*, *work*, and *employment* have specific and distinct meanings in this article. Employment refers to the contractual relationship between the employer and employee, and this is our central focus. Work refers to what the worker does, and *work quality* concerns the nature of tasks and the physical and social environment in which the work occurs. Jobs are a broader term capturing the combination of work and employment.

## JOBS AND HEALTH: SHIFTING FOCUS FROM WORK QUALITY TO EMPLOYEMENT QUALITY

The shifting nature of employment arrangements and labor experiences has challenged the adequacy of traditional approaches to investigating the relationship between work and health. The vast majority of occupational health studies have focused on work quality. Traditional occupational health research has focused on physical hazards, such as exposure to chemical agents or dangerous and physically demanding tasks or environments. This line of research has made tremendous contributions to general public health; for example, the International Agency for Research on Cancer, an agency within the World Health Organization, routinely evaluates occupational exposure as a basis for their human carcinogen designation. As economic activity in developed economies has moved away from industrial production and into service occupations, more attention has been directed at the psychological and social environment in the workplace. Since the early 1980s, job stress research has flourished, built on the fundamental premise that if the resources available to the worker are adequate for the demands in the workplace, then the health of the worker will be protected and enhanced; however, if the workplace demands overwhelm the worker’s resources, his or her health will be compromised (Karasek 1979). A significant body of literature has provided convincing evidence that support this premise (Daniels, Tregaskis, and Seaton 2007; Siegrist et al. 2007). Both lines of occupational health research—one focusing on physical, chemical, and biological hazards and the other on psychological and social work environment—assume that health risks arise from *work* tasks and environments, and thus have paid little attention to *employment* conditions.

In the EU over the last several decades, policy interest in improving the quality of jobs has been significant (Lisbon European Council 2000). This has driven empirical and theoretical research to identify high-versus low-quality jobs. Although no ultimate consensus has been established as to how to measure job quality, researchers agree on the reality of a conceptual distinction between work quality (the nature of tasks and work environment) and employment quality (the relational and contractual aspects of the employer-employee relationship) (Holman and McClelland 2011; Muñoz de Bustillo et al. 2009). Although workers experience both work quality and employment quality at the same time, by distinguishing the two, researchers can build on the large body of literature on work quality and health while clarifying the relationship between work quality and employment quality that may influence health. The distinction will help identify policy directions for protecting the health of working people.

## EMPLOYMENT QUALITY AS A MULTIDIMENSIONAL CONSTRUCT

To better understand health consequences of employment quality (EQ), we first need to recognize that the quality of employment is a multidimensional construct characterized by various conditions of the employer-employee relations. Several scholars have proposed ways to conceptualize EQ as a multidimensional construct (Holman and McClelland 2011; Muñoz de Bustillo et al. 2009). In this study, we build on a number of recent EU studies that have conceptualized EQ with the following seven dimensions: employment stability, material rewards, workers’ rights and social protections, standardized working time arrangements, training and employability opportunities, collective organization, and interpersonal power relations (Julià et al. 2017; Van Aerden et al.2014). These dimensions were drawn from a critical review of the employment quality literature with a specific focus on implications for worker well-being (Van Aerden et al. 2014), and thus together they capture various contractual arrangements and employment practices that employees experience.

A second important consideration is that jobs represent packages or configurations of different work and employment features, and that health implications stem from particular patterns in these features. One way to operationalize this is a typological approach, which identifies patterns of employment characteristics that holistically represent worker’s experience. Using the Standard Employment Relationship (SER)—permanent, full-time, regularly scheduled work with secure wages and benefits—as a reference point, we can characterize the experience of EQ with differences in the pattern of employment conditions across the seven EQ dimensions. For example, some jobs may offer a short-term contract, low pay, and too few hours; others may have too many work hours, high pay, and good benefits (Van Aerden et al. 2014). Although this is still a new approach, two European studies have reported significant association between EQ types and some general health indicators (Van Aerden, Gadeyne, and Vanroelen 2017; Van Aerden et al. 2016).

The typological approach complements the more traditional variable-based approach, which focuses on individual aspects of EQ (such as employment stability, work schedule, pay) separately and identifies their independent associations with health while assuming other aspects to be constant. Studies using the variable-based approach have linked non-standard employment—usually measured as perceived job insecurity or nonpermanent contract—to a variety of health outcomes, including increased injury rates and injury severity, musculoskeletal symptoms, and poor physical and mental health (Benach et al. 2014; Kim et al. 2012; Quinlan, Mayhew, and Bohle 2001; Silverstein et al. 1998). Poor health has been also associated with long working hours (O’Reilly and Rosato 2013; Virtanen et al. 2012), irregular and asocial work schedules (Jamal 2004; Martens et al. 1999), and mismatched preferences regarding working times (Wooden, Warren, and Drago 2009). Although they all suggest that components of EQ have potential health implications, being in disparate literatures and addressing only single aspects of EQ at a time, these findings have not formed a coherent approach for investigating health implications of the broader concept of EQ. Further, because poor employment conditions (for example, instability, irregular shift, low pay) tend to cluster in the same job, the variable-based approach is limited in its ability to illuminate health implications of poor EQ for workers, who experience jobs as a package.

## EMPLOYMENT QUALITY AND HEALTH

A theoretical underpinning for EQ’s health consequences is the fundamental cause theory of health (Link and Phelan 1995). It posits that money, knowledge, power, prestige, and social connections are personal resources that enable individuals to accumulate health advantages over time; hence the unequal access and distribution of these personal resources are fundamental causes of health inequalities. Most studies that apply this theory have used education as the proxy for the personal resources (see, for example, Masters, Link, and Phelan 2015). Recently, Emily Ahonen and her colleagues argued that jobs, with their complexity in providing both health-enhancing and damaging contexts throughout the adult life, influence the access and distribution of personal resources and thus are a crucial component in the application of fundamental cause theory (2018). According to this theory, EQ may affect health by influencing individuals’ access to money, knowledge, power, prestige, and social connections, which in turn shape their ability to accumulate health advantages over time. In the context of EQ, we operationalize these personal resources with three specific pathways that lead from EQ to health: material deprivation, stressors related to employment conditions, and occupational risk factors (Julià et al. 2017; Tompa et al. 2007).

The first pathway, material deprivation, involves whether the employment condition provides a worker sufficient income as well as non-wage material benefits (for example, health insurance, paid sick leave) to acquire necessities and health-enhancing goods. The association between income and health is well documented (Fritzell, Nermo, and Lundberg 2004). The mechanism is not only through access to necessities and goods, but also though psychosocial distress associated with deprivation such as low self-esteem (Gardner, Dyne, and Pierce 2004); poor satisfaction with jobs (Faragher, Cass, and Cooper 2005; Leigh and De Vogli 2016) and life in general (Cheung and Lucas 2015); and difficulties in long-term life planning (Bosmans et al. 2016; Julià et al. 2017; Tompa et al. 2007).

The second pathway is through employment-related stressors such as job insecurity and earning unfairness. If the employment contract is short term or hours fluctuate unexpectedly, workers will experience anxiety about keeping the job (job insecurity) and less control over their professional and personal lives, which may hinder career development, create powerlessness, and negatively affect family and other personal relationships. These effects are all associated with poor health (Clarke et al. 2007; Lewchuk, Clarke, and de Wolff 2008). Moreover, if two workers perform the same work tasks side by side but are paid differently because of their different employment conditions (for example, a SER secretary and a clerical worker sent from a temp agency), the sense of unfairness arises, which is also associated with poor health (Elovainio et al. 2010).

Finally, EQ may affect health through differential exposures to occupational risk factors. Even though work tasks are similar, workers under different employment conditions may be exposed to occupational hazards differently. SER workers, to whom the employer is committed long term, may receive thorough training, have opportunities to develop skills to perform tasks safely, and be able to change work processes so that they are safe. The employers are likely to be motivated to keep SER jobs safer because SER employee turnover is expensive. For non-SER workers (for example, short-term, substitute, subcontractors), employers may not invest many resources in their safety. Because of the power relations represented in employment conditions, some non-SER workers may be reluctant to refuse hazardous tasks (Arons-son 1999; Foley 2017; Quinlan, Mayhew, and Bohle 2001; Tompa et al. 2007). Besides occupational safety, job strain—a combination of high job demands and little control (Karasek 1979)—and workplace social support are robust predictors of health (de Lange et al. 2003; Thoits 2011). Employment conditions may influence the workers’ experience of both. Non-SER workers who are paid by the amount produced may have higher job demands than SER workers receiving hourly wages or salaries. Short-term contracts may not allow non-SER workers to form supportive connections in the workplace. All three mechanisms are conceptually plausible, but to date little systematic investigation has been done as to their importance in the relationship between EQ and health.

## THE CURRENT STUDY

Using data from the General Social Survey (2002–2014), we examine the association of EQ and three health indicators (self-rated health, mental health, and occupational injury) and explore three proposed mediating mechanisms (material deprivation, employment-related stressors, and occupational risk factors). Self-rated health is an indicator of broad health status (Idler and Benyamini 1997) and its significant association with EQ was reported previously in EU data (Van Aerden, Gadeyne, and Vanroelen 2017; Van Aerden et al. 2016). Mental health—also associated with EQ (Van Aerden, Gadeyne, and Vanroelen 2017; Van Aerden et al. 2016)—and occupational injury are more specific and contrasting health indicators. For mental health, material deprivation and employment-related stressors would be more salient mediating mechanisms, whereas traditional occupational risk factors would be more salient for occupational injury. Because our data are self-reported and cross-sectional, it is important to have contrasting health indicators so as not to capture completely spurious associations. Further, although the proposed mediating mechanisms are not competing hypotheses—rather, most likely all mechanisms are in effect simultaneously—the most salient mechanism may differ by the type of health consequence (for example, acute versus chronic) and by specific EQ features that distinguish a given employment condition from SER (for example, material rewards, employment stability, power relations). In this study, we investigate the linkages between EQ and health, as well as explore plausible mechanisms deserving of future investigation in this emerging field.

## DATA AND METHODS

This study uses data from the General Social Survey (GSS). The GSS is a nationally representative, repeated cross-sectional survey of non-institutionalized American adults conducted in face-to-face personal interviews by the National Opinion Research Center (Smith et al. 2013). In 2002, 2006, 2010, and 2014, the GSS included a module on the Quality of Work Life (QWL), which assessed an assortment of employment conditions among employed GSS respondents. This module was developed in collaboration with the National Institute for Occupational Safety and Health and with advice from a panel of experts in organizational behavior, occupational safety and health, and human resource management. A total of 5,961 respondents, pooled across the four survey years, completed the QWL module and indicated that they were currently employed (either in full- or part-time work, or temporarily not working due to strike, vacation, or temporary illness). From this sample, exclusion criteria were applied at two stages of our analysis: first, prior to latent class analysis to determine EQ categories, and, second, prior to regression analyses with health outcomes. All analyses are adjusted for survey sampling probabilities that account for number of adults in the household and nonresponse. Year-specific response rates for the GSS were between 70.1 percent and 71.4 percent.

### Construction of an Employment Quality Typology

The primary independent variable, a typology of EQ, was constructed by latent class analysis (LCA), which identifies mutually exclusive and exhaustive EQ types based on patterns of EQ indicator responses. In the GSS, we identified eleven indicators of EQ conditions that represent the seven dimensions of EQ described earlier (see table A1).^1^ The conceptualization and choice of EQ indicators is based on an established framework (Julià et al. 2017), and indicators we used from the GSS are similar to prior studies of EQ in Europe (Van Aerden, Gadeyne, and Vanroelen 2017; Van Aerden et al. 2014, 2015, 2016). LCA modeling in this study was conducted using the mixture modeling function with maximum likelihood (ML) estimation, including sample weights provided by the GSS, in Mplus version 8 (Muthén and Muthén 2010). Missing values were modeled with ML estimation assuming missing at random (Little and Rubin 2014).

In constructing EQ categories, we evaluate wage earning and self-employed worker populations separately: these employment arrangements are fundamentally different such that we expect the meaning of some EQ indicators to be dissimilar across the two groups (for example, mandatory overtime could be self-imposed for self-employed workers). Self-employment status was determined using the item “Are you self-employed or do you work for someone else?” Respondents with no information on self-employment were excluded (n = 5). We further excluded respondents without information for at least two EQ indicators (n = 23), retaining as many respondents as possible that contributed EQ items for the LCA. The final sample included in LCA modeling was 5,933 workers (n = 5,125 for wage earners, n = 808 for the self-employed).

Analyzing the wage earning and self-employed groups separately, we increased the number of classes stepwise and then selected the best LCA models through a two-step procedure that includes assessment of formal fit indices and a substantive interpretation of EQ types. Three model-fit indices, Bayesian Information Criteria, Akaike Information Criteria, and Vuong-Lo-Mendell-Rubin likelihood ratio test, indicate that the optimal solution within the wage earner sample was between four and seven EQ categories (see table A2). After taking into account the conceptual meaning of each measurement model by examining conditional response probabilities—that is, the within-class distributions of each response category—we chose a six-class model as the most meaningful (see table A3). In the self-employed sample, both fit indices and substantive interpretation indicated the two-category solution was best (see tables A2 and A4). Therefore, based on a combination of model fit and interpretation, eight EQ categories are identified as the most stable and meaningful solutions, six employment types within wage earners and two within self-employed workers. Further, an evaluation of LCA output show a clear pattern of nonrandom distribution of category-specific item response probabilities. This suggests that each of the included indicators possess predictive power for determining membership into the EQ types (Flaherty 2002)

We labeled the eight EQ types based on the probability of endorsing particular responses that distinguish one EQ type from another (see tables A3 and A4). These labels are meant to reflect the characteristic employment conditions that together create the workers’ experience of employment in each of the EQ types (see table 1). In addition to the SER-like type, the portfolio and precarious job types identified in the GSS are similar to those seen in prior studies of the EU labor market, and thus these labels are adopted in this article (Van Aerden, Gadeyne, and Vanroelen 2017).

#### Health Indicators

Given the multitude of potential manifestations of poor health associated with low-quality employment (Benach et al. 2014; Kim et al. 2012), and our expectation that the health consequences and mechanisms of EQ may vary depending on the patterns of employment conditions one is exposed to, we explore the relationship between EQ and three broad indicators of health. First, we examine self-rated health (SRH), measured by the standard question: “In general, would you say your health is excellent, very good, good, fair or poor? (fair/ poor = 1, good/very good/excellent = 0).” The SRH measure has strong predictive validity of mortality and morbidity (DeSalvo et al. 2006; Idler and Benyamini 1997; Singh-Manoux et al. 2007). Second, we assess frequent mental distress (FMD), measured using the general mental health item from the Centers for Disease Control and Prevention (CDC) four-item health-related quality of life index (HRQOL-4): “Now, thinking about your mental health, which includes stress, depression, and problems with emotions, for how many days during the past thirty was your mental health not good?” FMD is defined as fourteen or more mentally unhealthy days and is commonly used as a proxy for poor mental health in population health surveillance (Brown et al. 2003; CDC 1998, 2000, 2004). Last, we examine work-related injury. The number of injuries a respondent has experienced at work are assessed with the following question: “In the past twelve months, how many times have you been injured on the job?” The injury measure includes a count of injuries from zero to six and seven or more.

### Measures of Sociodemographic Characteristics

We adjust health outcomes models for five sociodemographic characteristics. Demographic variables included are sex (male, female), race-ethnicity (non-Hispanic white, non-Hispanic African American, Hispanic, other), nativity (born in the United States, born outside the United States), and age. Age is trichotomized into three groups corresponding with three meaningful periods in a working career: lift-off (younger than thirty), a mid-career period (thirty to fifty), and the end-of-career period (fifty-one and older) (Vanroelen et al. 2010). Educational attainment is included as less than high school, high school, associate degree, bachelor’s degree, and graduate degree. In this study, these variables are hypothesized to confound the EQ-health association: each predict labor market position and are associated with physical and mental health status.

### Measures of Potential Mediating Factors

We use the rich information on employment conditions available within the QWL to examine potential mediating mechanisms in the EQ-health association. To examine the first path-way, *material deprivation,* we use inadequate income, “Do you feel that the income from your job alone is enough to meet your family’s usual monthly expenses and bills” (no = 1, yes = 0) and inadequate fringe benefits, “My fringe benefits are good” (not too or not at all true = 1, very or somewhat true = 0). Second, we assess two indicators of an *employment-related stressors* pathway: job insecurity and earnings unfairness. Perceived job security is measured as degree of agreement with the statement “The job security is good” (not too or not at all true = 1, very or somewhat true = 0). Unfairness of earnings is measured with the question “How fair is what you earn on your job in comparison to others doing the same type of work you do” (much less than deserved = 1, somewhat less, about as much, somewhat more, or much more = 0). This is a distinct construct from inadequate income, though the two may be correlated, because the earning fairness is asked as social comparison whereas inadequate income was asked as a comparison with one’s needs. The third pathway, *traditional occupational risk factors,* is represented with three variables: job strain, high physical exposure, and low social support. Job strain was constructed from three items on job control (learn new things, variety, allows own decisions) and three items on job demands (work fast, enough time, no excessive work) all from the Job Content Questionnaire, specifically designed for job strain (Karasek et al. 1998). Each set of items were summed, split at the sample median score, and made into a quadrant: low-strain jobs (low demand and high control), high-strain jobs (high demand and low control), active jobs (high demand and high control), and passive jobs (low demand and low control) (Karasek et al. 1998). A dichotomized measure of high physical exposures combines two items asking whether a respondent’s job regularly requires forceful hand movements or awkward positions and repeated lifting, pushing, pulling, or bending (both present = 1, one or neither present = 0). The third occupational risk factor mediator is low levels of social support, which is commonly studied alongside job strain as a factor that moderates negative impacts of job strain (de Lange et al. 2003). This measure is constructed by combining four items: two measures of coworker support (such as “The people I work with take a personal interest in me”) and two measures of supervisor support (such as “My supervisor is helpful to me in getting the job done”). The social support variable is dichotomized such that high support is coded as a minimum average response of somewhat true or better, and is otherwise coded as low support.

### Statistical Analysis

In the GSS sample of wage earners and self-employed workers, relatively little data are missing in each variable included in regression analyses: only earnings unfairness (3.1 percent) and workplace social support (5.6 percent) variables had more than 2 percent missing. Because we did not have a theoretical basis for imputing these values from available GSS data, respondents who did not provide information on earnings fairness or social support were excluded from the analysis. Those who had missing data on other variables—that is, in order of most to least missing data (all less than 2 percent): job strain, benefits adequacy, job security, income adequacy, FMD, physical hazards exposure, occupational injury, SRH, and age—were also excluded. Due to the large number of variables and a high degree of non-overlapping missingness, the exclusion steps reduced the total weighted sample by 9.5 percent (n = 5,480). The final sample characteristics are presented in table 2. Respondents removed from the analysis were older, more likely to be born outside the United States, and reported less FMD than the analysis sample (see table A5). The proportion of removed respondents also varied by survey year; in addition to general concerns of secular trends, this provided further rationale for adjusting all regression models for year to account for potential survey effects.

To examine the relationship between EQ and health, as well as potential mediators of this relationship, we use Poisson regression with a robust error variance. The robust Poisson approach provides efficient and reliable estimates of a ratio measure of effect when the outcome measure is common and odds ratios overestimate risk (Coutinho, Scazufca, and Menezes 2008; Zou 2004). Model parameters are exponentiated to the ratio scale for presentation. For binary outcomes (SRH and FMD), the results of the robust Poisson are interpreted as prevalence ratios; for count data (injuries in last year), coefficients represent rate ratios. We conducted all regression analyses in r (Version 1.1.423) using the glm2 package (Marschner 2011); all data are included in the models with GSS survey sample weights, and robust 95 percent confidence intervals are calculated from Huber-White standard error estimates determined by the sandwich r package (Zeileis 2004).

The EQ typology is introduced into the analyses as each respondent’s estimated probability of membership into the eight job types. Estimates from the wage earner and self-employed LCA models are combined so that each respondent is assigned eight scores between 0 and 1, which add to 1 (self-employed workers have zero probability of membership in the six EQ types identified in wage earners, and vice versa). This approach reduces classification errors relative to modal assignment (classification into a single, most likely class), as the latent class probabilities inherently include information regarding the uncertainty of classifying individuals to a specific category (Hagenaars and McCutcheon 2002).

Evaluating each health indicator separately, we build a sequence of regression models: a basic model with EQ and survey year only, a model additionally controlled for demographics (age, sex, race-ethnicity, and nativity), and a model that additionally controls for education. The SER-like job type is used as the reference category for all analyses. Thus, the effect estimates describe the ratio of outcome occurrence with 100 percent probability of belonging to a particular EQ type compared with the out-come occurrence with 100 percent probability of belonging to the SER-like job type (Van Aerden, Gadeyne, and Vanroelen 2017; Van Aerden et al. 2016).

To examine the three mediating mechanisms, we followed the mediation test principles recommended by Reuben Baron and David Kenny (1986). That is, we first establish the association between EQ and all of the mediating variables (having examined the EQ-health association in our primary analysis), and then continue our nested regression analyses: a model with each of the three sets of mediating variables, and a model with all mediation variables. Log-likelihood ratio tests are conducted to assess the overall significance of EQ as well as model improvements as we include additional variables. Evidence for mediation is identified when EQ coefficients have a smaller magnitude or less statistically significant relationship with the health outcomes when mediator variables were introduced relative to the regression equation in which mediator variables were not introduced.

## RESULTS

In summarizing our findings, we first detail the relationship between EQ and our three health outcomes, including self-reported health, frequent mental distress, and work injury. Next, we describe associations between EQ and indicators of material deprivation, employment-related stressors, and occupational risk factors, operationalized here as potential mediating mechanisms of the EQ-health relationship. Finally, we report on exploratory analyses examining if the associations between EQ and health outcomes are explained by the proposed mediators.

### Association Between EQ and Three Health Indicators

EQ and the three health indicators were significantly associated in the basic model (that is, adjusted for survey year only), and additional adjustments for age, sex, race-ethnicity, nativity, and education did not substantively affect the associations (see tables A6 through A8). The associations of EQ with SRH, FMD, and work injury, adjusted for these demographic characteristics, are presented as model 1 in tables 3, 4, and 5, respectively. Compared with SER-like jobs, portfolio job holders were less likely to report poor SRH. Inflexible skilled job holders reported worse FMD and more work injuries. Dead-end and precarious job holders were more likely to report poor SRH, poor FMD, and more injuries. In contrast, optimistic precarious job holders were not different from SER in any of the health indicators. The two types of self-employed jobs did not differ from SER-like in SRH, but respondents in both skilled contractor and job-to-job types reported more injuries, and those from the job-to-job type also reported worse mental health.

### Association Between EQ and Proposed Mediating Variables

Before presenting mediation results, we first examine the associations of EQ types and proposed mediating variables (see table 6). All these variables are coded in the direction of health compromising. Portfolio job holders had lower levels of material deprivation and traditional occupational hazards than SER-like job holders, and did not differ significantly on employment-related stressors. Self-employed skilled contractors had a similar profile to portfolio job holders but were more similar to SER-like job holders in fringe benefits and social support. One difference is the higher exposure to physical hazards among skilled contractors than the SER-like type. Inflexible skilled job holders are similar to skilled contractors but are more likely to perceive unfairness in earnings compared with SER. Dead-end, precarious, and job-to-job types are similar in that they have higher levels of material deprivation, employment-related stressors, and occupational risk factors relative to SER-like jobs. Optimistic precarious jobs are distinct from any other EQ types in that despite high levels of material deprivation and job insecurity, they are similar to SER-like jobs in terms of fair earning and occupational risk factors. Taken together, the different patterns of associations between EQ types and mediating variables generally suggest health-protecting features in portfolio and skilled contractor jobs; health- damaging features in dead-end, precarious, job-to-job, and optimistic precarious jobs; and a complex combination of each for inflexible skilled jobs.

### Exploration of Potential Mediating Mechanisms

The results of regression models that include the mediating variables are presented in tables 3 through 5. When included in the EQ-SRH models, the material deprivation variables are associated with higher likelihood of reporting poor SRH, inadequate fringe benefits having a more robust association (see table 3, model 2). Inclusion of material deprivation variables resulted in slightly attenuated associations in some EQ types. In particular, our results suggest that dead-end and precarious jobs’ higher likelihood of reporting poor SRH, as well as portfolio jobs’ lower likelihood, may be explained by different levels of material deprivation experienced by those job holders. Employment-related stressors were strongly associated with poor SRH (model 3). When these mediators were included, associations for dead-end and precarious jobs were attenuated. Traditional occupational risk factors were also strongly associated with poor SRH (model 4), (and when these were included, associations for dead-end and precarious jobs were again attenuated. This suggests that both employment-related stressors and occupational risk factors may also explain the significant associations of EQ with SRH in dead-end and precarious job types. Finally, model 5, in which all mediating variables are included, shows large attenuation of all EQ associations. The association between EQ as a whole and SRH is also slightly diminished (that is, the *p*-value for the log-likelihood ratio test for EQ changed from <.001 to .049). Some mediators also show diminished associations with poor SRH, which indicates they are likely correlated with each other. Models with mediator variables are all significantly better at explaining the outcome variance than model 1.

Results for poor mental health (FMD) are shown in table 4, models 2 through 5. The material deprivation model (model 2) shows some attenuation in EQ coefficients from model 1 and the most pronounced attenuation in the precarious type. In the employment-related stressor model (model 3) and particularly the occupational risk factor model (model 4), we see attenuation of EQ coefficients for inflexible skilled, dead-end, and precarious types. Notably, precarious and job-to-job types consistently show higher likelihood of reporting poor mental health when individual sets of mediators are included. However, when all mediators are included in the model (model 5), all coefficients for EQ types reduced their magnitude from model 1, and EQ as a whole is no longer significantly associated with FMD (*p* = 0.285). Together with the observation that the mediators had strong and significant associations with FMD in expected directions in all models, model 5 finding suggests that these mediating variables may play an important role in the EQ-mental health association.

Table 5, models 2 through 5 present potential mediation in the EQ association with occupational injuries. In general, EQ’s association with occupational injuries did not change as much as it did with other outcomes when mediator variables were included in the model. Also the mediator variables are not as strongly associated with this outcome, aside from physical hazards exposure and low social support (components of occupational risk factors). The most striking difference from model 1 can be seen in model 4, in which traditional occupational risk factors were included as mediators. The coefficients for inflexible skilled, dead-end, and precarious jobs—the highest likelihoods of reporting occupational injuries in model 1—diminished drastically in model 4, and more modest attenuation was seen for skilled contractors and job-to-job. When all mediators were included (model 5), EQ’s association with occupational injury was similar to what we saw in model 4. Inflexible skilled jobs and dead-end jobs constantly had significantly higher likelihoods of reporting injuries compared with SER-like jobs, suggesting some other mechanisms are in effect.

## DISCUSSION

In this study, we examine the association between EQ and three indicators of health: general health, mental health, and occupational injury. Overall, we find significant associations between some EQ types and each of the three health indicators when compared with SER-like jobs after adjusting for sociodemographic characteristics. This study is part of a growing trend within occupational health research to expand its framework to consider the relational and contractual aspects of employment that affect health. A primary strength of this analysis is that EQ is measured using a multidimensional, typological approach, such that the EQ-health associations we find reflect health implications of employment as a package, rather than each aspect of employment. Another contribution of this study is an initial exploration of three possible mediating mechanisms between EQ and health, with these data generally supporting their plausibility.

### EQ Types and Health

The eight EQ types in our study had distinct associations with health. As expected, dead-end and precarious job holders had consistently higher likelihoods of reporting poor general and mental health as well as occupational injury. These EQ types are characterized by an accumulation of several unfavorable employment conditions, including high workplace harassment and low opportunity to develop, control over schedule, and employee involvement (see table A3). Also, dead-end and precarious job holders were similar in their experience of three mediating mechanisms: high levels of material deprivation, employment-related stressors, and occupational risk factors. Yet these two job types differed across several dimensions of EQ, including indicators of stability, material rewards, working time arrangements, and collective organization. To protect the health of workers in these EQ types, we need to investigate more purposefully the specific combination of employment conditions and work quality they experience as a package.

Likewise, inflexible skilled job holders and job-to-job workers also had worse mental health and injury experience than SER-like job holders. The two, however, represent clearly distinct combinations of EQ conditions: inflexible skilled jobs resemble stable, relatively well-paid employment but with excessive and inflexible hours; job-to-job workers experience a highly nonstandard employment arrangement with low pay and relatively low hours. They also starkly differ in their experience of the mediating mechanisms: inflexible skilled workers reported similar profile with SER-like workers except for higher physical hazards and unfair earnings; job-to-job workers reported all unfavorable experiences except for job strain. These differences, both in EQ characteristics and pro-posed mediating mechanisms, suggest that their poor health is a manifestation of distinct combinations of employment and working conditions that may warrant different approaches for intervention.

Unlike Karen Van Aerden and her colleagues (2016), who report high health risks for portfolio jobs from EU data, in our data portfolio jobs were generally not different from SER in terms of health. The U.S. portfolio jobs we identified are similar to European—characterized by generally the most favorable employment conditions—with one exception: the U.S. portfolio job holders did not suffer from mandatory extra days of work, whereas a defining feature of the EU portfolio jobs was uncompensated exceptional working times (Van Aerden et al. 2016). Portfolio job holders in our study reported a higher sense of material resource adequacy, fairness in their earnings and security in their jobs, and lower levels of occupational hazard exposures than SER-like. In European contexts, these relationships may be different.

Somewhat unexpectedly, optimistic precarious job holders did not differ from SER-like job holders on any of the three health indicators. This EQ type is characterized as very destan-dardized: that is, having the lowest hours, very low income, and highest probabilities of both irregular hours and nonpermanent arrangements within wage earners. Likewise, these job holders report higher levels of material resource inadequacy and job insecurity. Yet, these jobs also have an overall profile that includes several favorable EQ conditions, including relatively high schedule control, development opportunity, and employee involvement in decision making, suggesting the possibility that these workers are opting in to these types of jobs. Indeed, despite low pay and feelings of inadequate income, their sense of earnings unfairness is not different from SER-like job holders. This would generally comport with a recent study of Italian workers that found workers in nonstandard employment arrangements are a heterogeneous group and that voluntariness into these jobs was relevant to health status (Pirani 2017). Our finding of similar health to SER-like jobs suggests these workers may have other sources of health-protecting resources.

The two classes identified among the self-employed are quite different from each other. Skilled contractors resemble a highly paid, independent workforce, similar to portfolio job holders but engaged in jobs with time-specific contracts. Job-to-job workers have low pay and hours, with little involvement, and generally seem to have the weakest attachment to the labor market—although the extent to which this is by choice is uncertain, as they also possess flexibility and development opportunities. In occupational safety and health studies, self-employment has been understudied (Stephan and Roesler 2010), and if it is addressed, the heterogeneity among the self-employed has been neglected. Our findings indicate that there may be important differences among working people who self-identify as self-employed. Our study finds job-to-job workers to report poor mental health and yet the pro-posed mediators do not seem to explain the relationship. Because our sample sizes for the self-employed workers were limited (n ~800), these intriguing results need additional exploration with specific focus on self-employment.

### The Value of a Multidimensional, Typological Approach and Policy Implications

In conceptualizing EQ as a multidimensional construct, we believe that we are better able to capture key dimensions of workers’ employment experience that affect health and wellbeing. This approach also has potential to inform policymakers to enhance worker health through improved job quality. It is important to highlight that researchers in sociology, economics, and public health have struggled to conceptualize and measure EQ. Some researchers have focused too narrowly, especially on single dimensions such as employment arrangement or wages; others have attempted to include more nuance in their conception of poor- or low-quality jobs, only to find these conceptions quickly become too difficult to use in empirical analysis of actual working populations. A widely studied such concept is precarious employment, which can be defined generally as an accumulation of many unfavorable employment features (Julià et al. 2017). Deeply rooted in the tradition of sociological and labor relations literatures (Arnold and Bongiovi 2013; Kalleberg and Hewison 2013), the concept of precarious employment has been applied across analytical levels (for example, precarious employment, precarious work, precarious workers as a social class) and tends to have different meanings in different contexts (Burchell et al. 2014; Campbell and Price 2016). The development of specific scales to measure precarity is an active area of research (Lewchuk et al. 2014; Vives et al. 2015); however, even these approaches assess employment conditions using an aggregate scale ranging from low to high rather than something more dynamic. The LCA approach we used allows for conceiving of jobs as packages of employment features, and thus facilitates the conceptualization that health consequences of EQ will depend on specific patterns of features to which one is exposed.

The advantage of a typological approach, relative to dimensional approaches (that is, focusing on aspects of EQ separately), is its emphasis on the structure and distribution of simultaneously occurring employment conditions (Bergman and Magnusson 1997). In other words, a typological approach can identify profiles of risks for various segments of the labor force, which can be useful for policymakers to develop comprehensive interventions (Vanroelen et al. 2010). Dimensional approaches investigate specific features of employment conditions while assuming that all other aspects are constant. Thus, although potentially useful in identifying risk factors, resulting findings would suggest that policymakers effect narrowly focused interventions. Such interventions may improve job quality for some but may have no impact on others—or possibly even produce worse conditions for others. For example, based on research showing correlation between long work hours and poor health, one might propose limiting working hours to improve health. However, to cover the excess hours previously worked by permanent full-time employees, employers may create part-time jobs with unpredictable and inadequate hours. A typological approach would encourage policymakers to address unpredictable and inadequate hours, as well as inadequate pay, as a package. We believe that our approach is meaningful because it addresses a general picture of current U.S. labor market practice and the holistic experiences of American workers engaged in different types of employment.

The quality of one’s employment is modifiable through both policy levers and employer-driven workplace modifications. Overall, our findings suggest that if EQ conditions could be modified to resemble more closely the standard model of employment, many workers might experience better health. One example of an ambitious policy agenda can be found in the EU’s attempt to secure “more and better jobs” (Lisbon European Council 2000). As our exploration of mediating mechanisms suggests, the health-enhancing process may be through adequate material resources, fair earnings and job security, and lower exposures to occupational risk factors. If they are indeed mediating the EQ-health relationship, then changing these conditions may also help protect the health of working people. More generally, workplace policies can effectively redistribute resources to reduce inequality (for example, secure scheduling redistributes power from employers to workers) and can benefit all workers regardless of their personal resources or behaviors. Although employment conditions have received less attention than other aspects of socioeconomic position, such as education and income, the modifiable nature of employment makes it a critical determinant of health deserving of further consideration in both research and policy realms. Further, by specifically delineating between concepts of employment quality versus work quality, the EQ concept can be used to supplement and complement policy efforts to improve job quality as a whole.

### Limitations of This Study and Future Research Directions

A major limitation of this study is its reliance on self-reported cross-sectional data. The cross-sectional nature of the GSS data means that reverse causation—that is, poor health contributes to selection into jobs with poor employment conditions—cannot be ruled out as a possible explanation. In terms of self-reported measures, it would be ideal to obtain EQ indicators directly from employment records to overcome some of the inherent bias in self-reported data. Additionally, better (that is, more objective) measures of health outcomes would eliminate some of the bias found in these metrics. In particular, we found stronger mediation in associations between EQ and poor mental health, which may be inflated because these are especially sensitive to the person’s mental state at the time of data collection; for example, a worker in a poor mental health state may be more likely to perceive their EQ conditions as negative or poor than another worker in a better state of mental health (Conway and Lance 2010). Another limitation in this study is unmeasured confounding. For instance, unobserved factors such as early-life health, social support outside of the job, or local economic and policy contexts may confound the EQ-health association, potentially biasing effect estimates. However, the patterns of associations across health indicators give us some confidence that the observed associations are not artificial.

Another data-related limitation is the exclusion of a sizable portion (9.5 percent) of the overall GSS QWL sample in our regression analyses due to missing information. The majority of missingness occurred in covariates associated with our hypothesized mediation mechanisms; therefore, it is possible (but not likely) that if missing information on these variables is associated with other confounding characteristics, it could bias our results. As a crude sensitivity analysis, we repeated all regression models excluding only those with missing data required for the specific model; these exclusions showed no effect on our findings.

As for measure of EQ typology, EQ indicators included here are limited to those available within GSS data; in particular, detailed information on nonwage benefits, workers’ rights, and employability opportunities are lacking. Yet the GSS QWL module is among the richest individual-level data pertinent to EQ characteristics and health, and allows for an initial exploration of this construct in the United States. Further, we do not believe that having more indicators related to certain EQ dimensions is a problem for our LCA-based approach. This is primarily because each indicator represents a distinct aspect of the EQ construct. For instance, number of hours worked, when one works, and how much schedule flexibility one has each represent different facets of working time arrangements and power dynamics. Indeed, we find little evidence that EQ indicators are strongly correlated with each other, based on several statistical tests we conducted for association of categorical variables. In other words, rather than risking “overweighting” certain EQ dimensions, our LCA approach is able to identify heterogeneity within the diverse range of employment configurations seen among U.S. workers. Nevertheless, surveys need to better characterize both EQ and the health consequences of different occupational settings. The National Academy of Sciences recently called for improved surveillance of work-related exposures and health, including methods to include workers in nonstandard employment arrangements and other under-represented working groups (2018).

The mediation analysis we present is exploratory. Here we attempt to lay out our conceptual understanding of how EQ affects health, which has been rarely explored in the public health literature. In our study, EQ was associated with all of the variables representing proposed mediating mechanisms. When the mediators were included in the models, most had significant associations with the outcome variable in expected directions, and most EQ-health associations were attenuated. This supports that the hypothesized mediating processes linking EQ and health are plausible, and each mediation mechanism suggests a potential avenue for intervention. However, before concrete recommendations can be formed, more rigorous investigation with stronger study design must be pursued. Although we posit a strong conceptual rationale that EQ is antecedent to the evaluated mediators (for example, job insecurity, workplace social support, and the like would arise from one’s current job rather than contributing to selection into that job), the GSS data do not provide definitive empirical support. Thus, these results should be seen as suggestive evidence that the mechanisms proposed are useful. As this area of research continues to develop, we anticipate more suitable longitudinal data will become available for investigating the mediation questions of interest.

Despite these limitations, to our knowledge, this is the first study to rigorously evaluate a multidimensional construct of EQ to examine associations with employee health in the U.S. context. Although some studies have started to report multidimensional EQ and health relationships, they have been mostly restricted to Europe. The generalizability of European research to the U.S. context may be limited because of vast differences between the respective labor laws and regulation as well as social safety nets. These differences are reflected in the common finding that social class-based health disparities in Europe are less severe relative to those in the United States (Avendano et al. 2009; Avendano and Kawachi 2014). Fundamental causes theory suggests that the process of accumulating health advantages based on personal resources (that is, money, knowledge, power, prestige, and social ties) is firmly embedded in the dynamics of a given society (Masters, Link, and Phelan 2015). Because employment quality is likely to be an important part of this process, for EQ research to be useful in making changes, it needs to be embedded in the national context.

## CONCLUSION

The changing labor market has created new forms of employment that health researchers are not yet well equipped to investigate. Yet a long history of occupational health research makes us suspect certain combinations of employment features may contribute both to poor health of workers and to widening health inequalities in the society. It is therefore important to develop a conceptual framework and effective tools for investigating EQ from a public health perspective. This study is part of the emerging effort in this direction. Being exploratory in nature, this study generates many future research questions, some of which have been discussed above. Additional directions include replicating the EQ typology using different sources of U.S. data; exploring antecedents (especially socially determined characteristics) for workers to go into certain EQ types; and investigating at a macrolevel changes in EQ over time especially in relation to economic tides and population health in general. We argue that EQ should be recognized as a social determinant of health because of its complex and wide-reaching impacts on personal resources and chances for accumulating health advantages. We also argue that because of its complexity, EQ is better captured with a typological approach, as in this study, rather than in a variable-based approach of investigating single aspects separately. This approach can illuminate the clustering of disadvantages on the same segment of population, and potentially leads to policy-level solutions.

## Supplementary Material

Supplemental materials

## Figures and Tables

**Table 1.** Characteristics of Employment Quality Types Identified in the United States

Wage-Earner Types		Proportion of Overall Workforce

SER-like	Most similar to the Standard Employment Relationship (SER). These jobs have a very high probability of permanent, regular arrangement, full-time hours, adequate wages, working during the day shift, and have adequate information or equipment to complete work. Further, they have low probability of negative EQ conditions, such as excessive work hours, workplace harassment, or a lack of opportunity to develop.	22.2
Portfolio	Very high stability, pay, schedule control, opportunity, and strong power relations, but with long hours. Relative to all other types, these jobs have the highest probability of a permanent arrangement, high income, schedule control, employee involvement, and development opportunity, and low probabilities of experiencing harassment. These jobs also have a high probability of long work hours.	14.9
Inflexible skilled	Highly paid and involved class of workers, but with long and excessive work hours and little control over schedule. These jobs have high probability of high wages, opportunity to develop, union representation, and involvement in decision-making, but also high probability of irregular shifts, low schedule control, workplace harassment, long and mandatory extra working hours.	15.3
Dead-end	Stable, standard, full-time working arrangements with adequate wages, but with low opportunity and poor interpersonal and collective power relations. These jobs are mostly permanent, regular arrangements with middle-to-high wages, but with long and excessive work hours. However, these jobs are distinguished by having very low levels of development opportunity, schedule control, and employee involvement. They lack adequate information or equipment to perform job, and experience high workplace harassment. Counterintuitively, these jobs also have the highest union representation.	12.0
Precarious	Nonstandard working arrangements, low wages, lack of opportunity, and poor interpersonal and collective power relations. Compared to other wage-earner job types, these jobs have a high probability of nonpermanent working arrangements, low wages, part-time hours, and irregular shifts. Further, these jobs have low development opportunity, schedule control, union representation, and employee involvement, and experience high workplace harassment.	11.5
Optimistic precarious	Non-standard arrangements with low wages, but opportunity to develop and strong interpersonal power relations. These jobs are mostly similar to precarious job type, but distinguishing features are low probability of full-time hours and high levels of schedule control, employee involvement, and development opportunity. They also have lower experience of harassment at work.	10.5
Skilled contractor	High wages, opportunity to develop, and strong interpersonal power relations, but with nonstandard working arrangements and long and excessive hours. These jobs are mostly nonpermanent arrangements with long and excessive hours, and relatively high probability of irregular work times. These jobs also have high levels of schedule control, decision-making involvement, and development opportunity, accompanied by low levels of workplace harassment.	5.3
Job-to-job	Highly nonstandardized working arrangements with low income, but with opportunity to develop and strong interpersonal power relations. These jobs are predominately nonpermanent arrangements, with low income, few hours, and low union representation. The jobs also have high schedule control and opportunity to develop, and low harassment experience.	8.3

*Source:* Authors’ compilation based on General Social Survey (Smith et al. 2013).

*Note:* For additional information on EQ types, see tables A3 and A4.

**Table 2.** Characteristics of Sample Used in Regression Analysis (Weighted)

Characteristic	Level	Frequency (Percent)

n		5,480
Survey year	2002	1,659 (30)
	2006	1,579 (29)
	2010	1,075 (20)
	2014	1,166 (21)
**Sociodemographic characteristics**		
Age	Thirty or younger	1,342 (24)
	Thirty-one to fifty	2,621 (48)
	Fifty-one and older	1,518 (28)
Sex	Male	2,695 (49)
	Female	2,785 (51)
Race-ethnicity	White	3,889 (71)
	Black	728 (13)
	Other	233 ( 4)
	Hispanic	630 (11)
Nativity	U.S. born	4,811 (88)
	Not U.S. born	669 (12)
Highest degree	Less than high school	491 ( 9)
	High school	2,824 (52)
	Junior college	516 ( 9)
	Bachelor	1,083 (20)
	Graduate	566 (10)
**Health indicators**		
Self-reported health (SRH)	Good	4,755 (87)
	Poor	725 (13)
Frequent mental distress (FMD)	Absent	4,924 (90)
	Present	556 (10)
Work-related injuries in past year	0	4,882 (89)
	1	382 (7)
	2	99 (2)
	3 or more	116 (2)

*Source:* Authors’ compilation based on General Social Survey (Smith et al. 2013).

**Table 3.** Regression Analysis of Association of EQ and Self-Rated Health (SRH), and Inclusion of Potential Mediators

Independent Variable	Model 1 Estimate (95% Cl)		Model 2 Estimate (95% Cl)		Model 3 Estimate (95% Cl)		Model 4 Estimate (95% Cl)		Model 5 Estimate (95% Cl)	

**EQ typology (ref = SER-like)**		<0.001^a^		<0.001^a^		<0.001^a^		0.001^a^		0.049^a^
Portfolio	0.62 (0.39–0.97)	*	0.67 (0.43–1.05)		0.60 (0.38–0.95)	*	0.64 (0.41–1.02)		0.66 (0.41–1.04)	
Inflexible skilled	0.75 (0.50–1.12)		0.76 (0.51–1.13)		0.72 (0.49–1.07)		0.67 (0.45–1.01)		0.66 (0.45–0.99)	*
Dead-end	1.84 (1.31–2.57)	***	1.63 (1.16–2.29)	**	1.45 (1.02–2.06)	*	1.20 (0.84–1.72)		1.07 (0.74–1.55)	
Precarious	1.65 (1.15–2.37)	**	1.34 (0.92–1.93)		1.37 (0.95–1.97)		1.27 (0.87–1.85)		1.04 (0.71–1.53)	
Optimistic precarious	1.31 (0.90–1.89)		1.12 (0.77–1.63)		1.25 (0.86–1.80)		1.35 (0.94–1.95)		1.17 (0.81–1.70)	
Skilled contractor	1.13 (0.64–1.98)		1.17 (0.67–2.03)		1.12 (0.64–1.96)		1.09 (0.62–1.90)		1.09 (0.63–1.89)	
Job-to-job	1.03 (0.69–1.54)		0.90 (0.60–1.35)		0.92 (0.63–1.36)		0.99 (0.66–1.47)		0.85 (0.57–1.26)	
**Material deprivation**										
Inadequate income			1.14 (0.96–1.35)						1.07 (0.90–1.26)	
Inadequate fringe benefits			1.51 (1.28–1.78)	***					1.33 (1.12–1.59)	***
**Employment-related stressors**										
Unfair earning					1.32 (1.10–1.60)	***			1.17 (0.97–1.42)	
Job insecurity					1.64 (1.37–1.97)	***			1.43 (1.18–1.73)	**
**Traditional occupational risk factors**										
Job strain (ref = low strain)								<0.001^a^		0.002^a^
Active jobs							1.39 (1.11–1.75)	**	1.35 (1.07–1.69)	**
Passive jobs							1.19 (0.97–1.47)		1.18 (0.96–1.45)	
High strain jobs							1.65 (1.29–2.11)	***	1.56 (1.22–1.99)	***
High physical exposures							1.35 (1.14–1.59)	***	1.31 (1.11–1.55)	**
Lack of workplace social support							1.25 (1.05–1.49)	*	1.06 (0.88–1.28)	
AIC^b^	4252		4225		4216.5		4215.1		4185.8	
Log likelihood ratio test comparing each model with model l,*χ*^2^(*df*),*p*-value			*χ*^2^=31.04, *df*= 2, *p*<.001		*χ*^2^=39.47, *df*= 2, *p*<.001		*χ*^2^=46.94, *df*= 5, *p*<.001		*χ*^2^=84.18,*df*=9, *p*<.001	

*Source:* Authors’ compilation based on General Social Survey (Smith et al. 2013).

*Note:* Prevalence ratios and 95 percent confidence intervals are shown. All models are adjusted for age, gender, race, nativity, education, and survey year.

a *p*-value for the log likelihood ratio test.

b Akaike Information Criteria.

* *p* < .05;

** *p* < .01;

*** *p* < .001

**Table 4.** Regression Analysis of Association of EQ and Frequent Mental Distress (FMD), and Inclusion of Potential Mediators

Independent Variable	Model 1 Estimate (95% Cl)		Model 2 Estimate (95% Cl)		Model 3 Estimate (95% Cl)		Model 4 Estimate (95% Cl)		Model 5 Estimate (95% Cl)	

**EQ typology (ref = SER-like)**		<0.00 1^a^		<0.00 1^a^		0.003^a^		0.022^a^		0.285^a^
Portfolio	1.03 (0.60–1.75)		1.12 (0.66–1.91)		0.98 (0.57–1.66)		0.94 (0.55–1.60)		0.94 (0.55–1.61)	
Inflexible skilled	1.87 (1.20–2.91)	**	1.90 (1.22–2.94)	**	1.73 (1.12–2.67)	*	1.44 (0.92–2.26)		1.41 (0.91–2.20)	
Dead-end	2.76 (1.78–4.28)	***	2.45 (1.57–3.81)	***	1.95 (1.26–3.03)	**	1.46 (0.92–2.32)		1.28 (0.80–2.02)	
Precarious	2.59 (1.66–4.03)	***	2.06 (1.30–3.27)	**	1.91 (1.23–2.98)	**	1.83 (1.18–2.86)	**	1.45 (0.92–2.29)	
Optimistic precarious	1.58 (0.97–2.58)		1.35 (0.82–2.24)		1.48 (0.90–2.42)		1.68 (1.03–2.74)	*	1.49 (0.91–2.46)	
Skilled contractor	1.60 (0.79–3.25)		1.75 (0.86–3.56)		1.57 (0.78–3.17)		1.37 (0.70–2.68)		1.46 (0.75–2.87)	
Job-to-job	1.87 (1.16–3.03)	*	1.65 (1.01–2.68)	*	1.61 (1.03–2.53)	*	1.65 (1.04–2.64)	*	1.44 (0.91–2.27)	
**Material deprivation**										
Inadequate income			1.39(1.13–1.7)	**					1.26 (1.03–1.55)	*
Inadequate fringe benefits			1.35(1.1–1.65)	**					1.09 (0.88–1.35)	
**Employment-related stressors**										
Unfair earning					1.70 (1.38–2.10)	***			1.42 (1.15–1.76)	**
Job insecurity					1.74 (1.42–2.15)	***			1.44 (1.16–1.80)	**
**Traditional occupational risk factors**										
Job strain (ref = low strain)								<0.001^a^		<0.001^a^
Active jobs							1.95 (1.50–2.52)	***	1.82 (1.41–2.36)	***
Passive jobs							1.11 (0.85–1.45)		1.09 (0.84–1.42)	
High strain jobs							1.63 (1.20–2.22)	**	1.53 (1.13–2.08)	**
High physical exposures							1.26 (1.04–1.53)	*	1.22 (1.00–1.47)	*
Lack of workplace social support							1.68 (1.37–2.06)	***	1.42 (1.14–1.78)	**
AIC^b^	3531.5		3510.5		3476.2		3457.5		3429.2	
Log likelihood ratio test comparing each model with model 1, *χ*^2^(*df*), *p*-value			*χ*^2^=25.05, *df*=2, *p*<.001		*χ*^2^=59.31, *df*=2, *p*<. 001		*χ*^2^=84.05, *df*=5, *p*<.001		*χ*^2^=120.3, *df*=9, *p*<. 001	

*Source:* Authors’ compilation based on General Social Survey (Smith et al. 2013).

*Note:* Prevalence ratios and 95 percent confidence intervals are shown. All models are adjusted for age, gender, race, nativity, education, and survey year.

a *p*-value for the log likelihood ratio test.

b Akaike Information Criteria.

* *p* < .05;

** *p* < .01;

*** *p* < .001

**Table 5.** Regression Analysis of Association of EQ and Workplace Injuries, and Inclusion of Potential Mediators

Independent Variable	Model 1 Estimate (95% Cl)		Model 2 Estimate (95% Cl)		Model 3 Estimate (95% Cl)		Model 4 Estimate (95% Cl)		Model 5 Estimate (95% Cl)	

**EQ typology (ref = SER-like)**		<0.00l^a^		<0.00l^a^		<0.00l^a^		<0.00l^a^		<0.00l^a^
Portfolio	0.85 (0.42–1.71)		0.90 (0.45–1.81)		0.82 (0.41–1.66)		0.95 (0.47–1.90)		0.97 (0.48–1.94)	
Inflexible skilled	3.61 (2.04–6.39)	***	3.66 (2.07–6.48)	***	3.41 (1.92–6.05)	***	2.64 (1.46–4.79)	**	2.61 (1.43–4.79)	**
Dead-end	3.93 (2.21–7.00)	***	3.58 (2.02–6.34)	***	3.29 (1.77–6.11)	***	2.34 (1.31–4.18)	**	2.19 (1.20–3.99)	*
Precarious	2.30 (1.25–4.25)	**	1.91 (1.02–3.57)	*	1.95 (1.06–3.57)	*	1.55 (0.85–2.83)		1.34 (0.73–2.46)	
Optimistic precarious	0.97 (0.46–2.05)		0.87 (0.40–1.86)		0.95 (0.45–2.00)		1.06 (0.51–2.19)		0.99 (0.47–2.09)	
Skilled contractor	2.26 (1.03–4.96)	*	2.41 (1.11–5.24)	*	2.22 (1.02–4.83)	*	1.79 (0.82–3.91)		1.87 (0.86–4.04)	
Job-to-Job	2.12 (1.05–4.25)	*	1.93 (0.96–3.88)		1.98 (1.00–3.90)	*	1.70 (0.86–3.38)		1.60 (0.82–3.16)	
**Material deprivation**										
Inadequate income			1.28 (0.97–1.68)						1.17 (0.89–1.55)	
Inadequate fringe benefits			1.29 (1.00–1.66)	*					1.10 (0.83–1.45)	
**Employment-related stressors**										
Unfair earning					1.70 (1.28–2.25)	***			1.36 (1.01–1.81)	*
Job insecurity					1.12 (0.8–1.56)				0.96 (0.67–1.39)	
**Traditional occupational risk factors**										
Job strain (ref = low strain)								<0.00l^a^		<0.00l^a^
Active jobs							1.28 (0.90–1.83)		1.23 (0.87–1.75)	
Passive jobs							0.87 (0.64–1.18)		0.87 (0.64–1.18)	
High strain jobs							1.25 (0.85–1.84)		1.23 (0.84–1.82)	
High physical exposures							3.23 (2.35–4.43)	***	3.14 (2.29–4.30)	***
Lack of workplace social support							1.43 (1.10–1.86)	**	1.35 (1.02–1.79)	*
AIC^b^	6,663.4		6,633.1		6,614		6,290.9		6,269.7	
Log likelihood ratio test comparing each model with model 1, *χ*^2^(*df*), *p*-value			*χ*^2^=34.34, *df*=2, *p*<.001		*χ*^2^=53.46, *df*=2, *p*<.001		*χ*^2^=382.5, *df*=5, *p*<.001		*χ*^2^=411.7, *df*=9, *p*<.001	

*Source:* Authors’ compilation based on General Social Survey (Smith et al. 2013).

*Note:* Prevalence ratios and 95 percent confidence intervals are shown. All models are adjusted for age, gender, race, nativity, education, and survey year.

a *p*-value for the log likelihood ratio test.

b Akaike Information Criteria.

* *p* < .05;

** *p* < .01;

*** *p* < .001

**Table 6.** Relative Comparison of Prevalence of EQ-Health Mediators Within Each Employment Category Relative to SER-Like Jobs

	Material Deprivation		Employment-Related Stressors		Occupational Risk Factors		
	Inadequate Fringe Benefits	Inadequate Income	Job Insecurity	Unfairness of Earnings	High Strain Jobs^a^	High Physical Exposures	Low Social Support

EQ typology (ref = SER-like)							
Portfolio	Lower	Lower	n.s.	n.s.	Lower	Lower	Lower
Inflexible skilled	n.s.	Lower	n.s.	Higher	n.s.	Higher	n.s.
Dead-end	Higher	n.s.	Higher	Higher	Higher	Higher	Higher
Precarious	Higher	Higher	Higher	Higher	Higher	Higher	Higher
Optimistic precarious	Higher	Higher	Higher	n.s.	n.s.	n.s.	n.s.
Skilled contractor	n.s.	Lower	n.s.	n.s.	Lower	Higher	n.s.
Job-to-job	Higher	Higher	Higher	Higher	n.s.	Higher	Higher

*Source:* Authors’ compilation based on General Social Survey (Smith et al. 2013).

*Note:* Lower/Higher: statistically significant difference (*p*-value < 0.05) compared to *SER-like* jobs in Poisson regression with mediator as dependent variable (adjusted for survey year), n.s.: not statistically different from *SER-like* jobs.

a While the job strain measure contains four categories, only a dichotomous measure of high strain or not is tested for association with EQ in this analysis.

## Footnotes

1. See the online appendix (https://www.rsfjournal.org/content/5/4/258/tab-supplemental) for tables A1 through A8.
