# Supplementary material for: Evaluating Employment Quality as a Determinant of Health in a Changing Labor Market
Source: RSF. Author manuscript; Available in PMC 2020 Sep 1. (PMC6756794; doi:10.7758/RSF.2019.5.4.09)
Supplement: Supplemental materials [file NIHMS1032715-supplement-Supplemental_materials.pdf]

Table A1. Operationalization of indicators of employment quality within General Social Survey.

Dimensions	Indicators	Operationalization
[1] Employment stability	[1] Employment contract/arrangement	[1] Two categories: 1) regular, permanent employment; 2) non-permanent arrangement (independent contractor/freelancer; working under a contractor; employment on an on-call basis, or paid by temporary agency)
[2] Material Rewards	[2] Income level	[2] Year-specific sample quartiles, three categories: lowest, second or third, and highest quartile
[3] Workers' rights & social protection	[3] Mandatory extra days of work	[3] Three categories: 1) zero; 2) between 1 and 10; and 3) >11 days of required extra work hours
[4] Working time arrangements	[4a] Long working hours	[4a] Four categories: 1) <24 hours; 2) 25-36 hours; 3) 37-48 hours; and 4) >48 hours
	[4b] Working times regularity	[4b] Three categories: 1) day shift; 2) afternoon or night shift; and 3) split, irregular, on-call, or rotating shifts
[5] Employability opportunities	[5] Opportunity to develop abilities	[5] Dichotomous item indicating if worker has an opportunity to develop their own special abilities: yes ('very true', 'somewhat true') and no ('not too true', 'not at all true')
[6] Collective organization	[6a] Have adequate training, equipment, information	[6a] Combines two items asking if workers 1) have enough help and equipment, and 2) enough information to get job done. Dichotomized to yes ('often' or 'sometimes' responses for both of these items) and no (responses of 'rarely' or 'never' on at least one)
	[6b] Union representation	[6b] Dichotomous item indicating union membership: yes and no
[7] Interpersonal power relations	[7a] Employee involvement	[7a] Three categories: respondent is 1) 'often'; 2) 'sometimes'; and 3) 'rarely' or 'never' able to make decision on the job that affect them
	[7b] Control over schedule	[7b] Combines two items asking ability of workers to change starting/finishing times and to take off work for personal/family reasons. Three categories: 1) 'high control'; 2) 'medium control'; and 3) 'low control'
	[7c] Subjected to harassment/abuse	[7d] Combines two items indicating having been 1) sexually harassed or 2) threatened or harassed in any other way by anyone while at their job: yes ('yes' to either item) and no

Source: Authors' compilation based on General Social Survey (Smith et al. 2013).

Table A2. Comparison of model fit indices in LCA modeling of wage-earning and self-employed working populations to identify EQ.

# of classes	Log likelihood	AIC	BIC	VLMR-LRT
Wage-earner sample				
2	-39972	80023	80278	0.000
3	-39498	79114	79500	0.000
4	-39326	78810	79327	0.000
5	-39201	78600	**79248**	**0.002**
6	-39125	78488	79267	0.689
7	-39068	**78413**	79322	0.760
Self-employed sample				
2	-5748	11574	**11757**	**0.000**
3	-5692	11501	11778	0.828
4	-5654	11465	11836	0.761
5	-5617	11432	11897	0.761
6	-5588	11415	11973	0.764
7	-5564	**11406**	12059	0.760

Source: Authors' compilation based on General Social Survey (Smith et al. 2013).
Notes: AIC: Akaike Information Criteria. BIC: Bayesian Information Criteria. VLMR-LRT: Vuong-Lo-Mendell-Rubin likelihood ratio test. Bolding denotes lowest number of classes recommended by each fit indices.

Table A3. Distribution of conditional response probabilities across EQ types identified in wage-earner population

EQ indicator	Response category	Sample proportion	SER-like	Porfolio	Inflexible skilled	Dead-end	Precarious	Optimistic precarious
	Class proportion		0.257	0.139	0.134	0.172	0.121	0.177
**Employment arrangement**								
	Reg/permanent	0.894	0.933	0.950	0.900	0.941	0.864	0.706
	Non-permanent	0.106	0.067	0.050	0.100	0.059	0.136	0.294
**Income**								
	Lowest income quartile	0.275	0.164	0.037	0.119	0.078	0.823	0.769
	2nd/3rd income quartile	0.533	0.729	0.438	0.598	0.800	0.177	0.198
	Highest income quartile	0.193	0.107	0.525	0.282	0.123	0.000	0.033
**Mandatory extra days of work**								
	None	0.801	0.865	0.846	0.585	0.710	0.849	0.966
	1-10 days	0.137	0.135	0.091	0.227	0.173	0.135	0.034
	11+ days	0.062	0.000	0.062	0.188	0.117	0.017	0.000
**Working hours**								
	<24 hrs	0.103	0.010	0.014	0.034	0.029	0.200	0.504
	25-36 hrs	0.132	0.083	0.044	0.066	0.031	0.324	0.362
	37-48	0.506	0.907	0.418	0.257	0.634	0.440	0.071
	>48 hrs	0.258	0.000	0.524	0.644	0.305	0.035	0.063
**Working times regularity**								
	Day shift	0.734	0.877	0.924	0.620	0.715	0.530	0.572
	Afternoon/night shift	0.121	0.070	0.001	0.124	0.159	0.289	0.167
	Split/irregular/rotating	0.145	0.053	0.075	0.255	0.126	0.181	0.261
**Opportunity to develop abilities**								
	Very true, opportunity	0.359	0.356	0.602	0.524	0.047	0.151	0.359
	Somewhat true, opp	0.434	0.541	0.358	0.447	0.336	0.378	0.471
	Not true, opportunity	0.207	0.103	0.040	0.029	0.617	0.471	0.170
**Have adequate training, info, equipment**								
	Often/sometimes have	0.868	0.950	0.925	0.904	0.563	0.820	0.961
	Rarely/never have	0.132	0.050	0.075	0.096	0.437	0.180	0.039
**Union representation**								
	Union member	0.147	0.136	0.031	0.268	0.291	0.088	0.045
	Not union member	0.853	0.864	0.969	0.732	0.709	0.912	0.955
**Control over schedule**								
	High control	0.322	0.317	0.739	0.119	0.142	0.066	0.522
	Medium control	0.377	0.457	0.261	0.420	0.331	0.345	0.396
	Low control	0.301	0.226	0.000	0.461	0.527	0.589	0.082
**Employee involvement**								
	Often involved	0.397	0.400	0.639	0.554	0.175	0.165	0.324
	Sometimes involved	0.385	0.453	0.315	0.363	0.364	0.355	0.434
	Rarely/never involved	0.218	0.147	0.046	0.083	0.461	0.481	0.243
**Workplace harassment/threats**								
	Yes harass/threat	0.114	0.077	0.048	0.165	0.221	0.159	0.041
	No harass/threat	0.886	0.923	0.952	0.835	0.779	0.841	0.959

Source: Authors' compilation based on General Social Survey (Smith et al. 2013).

Table A4. Distribution of conditional response probabilities across EQ types identified in self-employed

EQ indicator	Response category	Sample proportion	Skilled Contractor	Job-to-job
	Class proportion		0.338	0.612
**Employment arrangement**				
	Reg/permanent	0.177	0.301	0.098
	Non-permanent	0.823	0.699	0.902
**Income**				
	Lowest income quartile	0.329	0.045	0.520
	2nd/3rd income quartile	0.357	0.359	0.355
	Highest income quartile	0.314	0.596	0.124
**Mandatory extra days of work**				
	None	0.771	0.596	0.883
	1-10 days	0.142	0.203	0.103
	11+ days	0.087	0.201	0.014
**Working hours**				
	<24 hrs	0.213	0.004	0.346
	25-36 hrs	0.160	0.052	0.229
	37-48	0.263	0.275	0.255
	>48 hrs	0.364	0.669	0.170
**Working times regularity**				
	Day shift	0.634	0.707	0.588
	Afternoon/night shift	0.026	0.000	0.042
	Split/irregular/rotating	0.340	0.293	0.370
**Opportunity to develop abilities**				
	Very true, opportunity	0.675	0.799	0.595
	Somewhat true, opp	0.233	0.201	0.254
	Not true, opportunity	0.092	0.000	0.151
**Have adequate training, info, equipment**				
	Often/sometimes have	0.934	0.968	0.912
	Rarely/never have	0.066	0.032	0.088
**Union representation**				
	Union member	0.030	0.026	0.033
	Not union member	0.970	0.974	0.967
**Control over schedule**				
	High control	0.629	0.563	0.671
	Medium control	0.270	0.321	0.238
	Low control	0.101	0.116	0.091
**Employee involvement**				
	Often involved	0.448	0.634	0.328
	Sometimes involved	0.254	0.242	0.262
	Rarely/never involved	0.298	0.123	0.410
**Workplace harassment/threats**				
	Yes harass/threat	0.072	0.074	0.070
	No harass/threat	0.928	0.926	0.930

Source: Authors' compilation based on General Social Survey (Smith et al. 2013).

Table A5. Comparing the sample used in regression analysis with excluded respondents (weighted).

		Analysis Sample	Excluded[a]	
**Characteristic**	**Level**	**Frequency (percent)**	**Frequency (percent)**	p-value[b]
n		5480	575	
Year	2002	1659 (30)	144 (25)	0.025
	2006	1579 (29)	193 (34)	
	2010	1075 (20)	129 (22)	
	2014	1166 (21)	109 (19)	
Sociodemographic characteristics				
Age	<=30	1342 (24)	107 (19)	<0.001
	31-50	2621 (48)	226 (40)	
	>51	1518 (28)	226 (40)	
Sex	Male	2695 (49)	286 (50)	0.802
	Female	2785 (51)	289 (50)	
Race/Ethnicity	White	3889 (71)	405 (70)	0.231
	Black	728 (13)	67 (12)	
	Other	233 (4)	37 (6)	
	Hispanic	630 (11)	66 (11)	
Nativity	U.S. born	4811 (88)	467 (81)	<0.001
	Non-U.S. born	669 (12)	108 (19)	
Highest degree	Less than HS	491 (9)	53 (9)	0.159
	High school	2824 (52)	270 (47)	
	Junior college	516 (9)	61 (11)	
	Bachelor	1083 (20)	112 (20)	
	Graduate	566 (10)	79 (14)	
Health indicators				
Self-reported health (SRH)	Good SRH	4755 (87)	467 (85)	0.399
	Poor SRH	725 (13)	81 (15)	
Frequent mental distress (FMD)	Absent	4924 (90)	485 (93)	0.026
	FMD	556 (10)	35 (7)	
Work-related injuries in past year	0	4882 (89)	494 (92)	0.142
	1	382 (7)	21 (4)	
	2	99 (2)	8 (2)	
	3 or more	116 (2)	10 (2)	

Source: Authors' compilation based on General Social Survey (Smith et al. 2013).
Notes: [a] Excluded from regression analyses due to missing covariate information. [b] Chi-square test: difference between variable responses in final sample vs. excluded respondents.

Table A6. Association between employment quality type and self-rated health (prevalence ratios).

	Basic Model		Demographics		Demographics & Education	
	Estimate (95% CI)		Estimate (95% CI)		Estimate (95% CI)	
EQ typology (ref. = SER-like jobs)						
Portfolio	0.50 (0.32-0.79)	**	0.49 (0.31-0.78)	**	0.62 (0.39-0.97)	*
Inflexible skilled	0.70 (0.47-1.05)		0.71 (0.48-1.06)		0.75 (0.50-1.12)	
Dead-end	1.87 (1.33-2.64)	***	1.83 (1.30-2.58)	***	1.84 (1.31-2.57)	***
Precarious	1.83 (1.28-2.61)	***	1.97 (1.39-2.81)	***	1.65 (1.15-2.37)	**
Optimistic precarious	1.33 (0.91-1.92)		1.39 (0.96-2.00)		1.31 (0.90-1.89)	
Skilled contractor	1.12 (0.64-1.96)		1.01 (0.58-1.79)		1.13 (0.64-1.98)	
Job-to-job	1.12 (0.75-1.67)		1.06 (0.70-1.59)		1.03 (0.69-1.54)	
Age (ref. = <30)						
31-50			1.35 (1.09-1.66)	**	1.35 (1.10-1.66)	**
>51			1.67 (1.34-2.09)	***	1.64 (1.32-2.05)	***
Female (ref. = male)			0.93 (0.79-1.09)		0.99 (0.84-1.16)	
Race/ethnicity (ref. = White)						
Black			1.21 (0.98-1.50)		1.12 (0.90-1.40)	
Other			1.29 (0.88-1.89)		1.38 (0.95-2.00)	
Hispanic			1.23 (0.93-1.62)		1.06 (0.79-1.42)	
Nativity (ref. = Born in U.S.)			1.05 (0.79-1.38)		1.03 (0.78-1.37)	
Education (ref. = less than HS)						
High school					0.62 (0.50-0.77)	***
Junior college					0.53 (0.38-0.73)	***
Bachelors					0.38 (0.28-0.52)	***
Graduate school					0.37 (0.26-0.54)	***
Intercept	0.11 (0.08-0.14)	***	0.08 (0.06-0.11)	***	0.13 (0.09-0.19)	***
AIC	4313.4		4297.2		4252	
Log-likelihood ratio test comparing each model with the previous one			χ^2=30.16, *df*=7, *p*<0.001		χ^2=53.20, *df*=4, *p*<0.001	

Source: Authors' compilation based on General Social Survey (Smith et al. 2013).
Notes: All models are adjusted for survey year. * p-value < 0.05; ** p-value < 0.01; *** p-value < 0.001.

Table A7. Association between employment quality type and frequent mental distress (prevalence ratios).

	Basic Model		Demographics		Demographics & Education	
	Estimate (95% CI)		Estimate (95% CI)		Estimate (95% CI)	
EQ typology (ref. SER-like jobs)						
Portfolio	0.76 (0.45-1.29)		0.81 (0.48-1.38)		1.03 (0.60-1.75)	
Inflexible skilled	1.70 (1.09-2.65)	*	1.75 (1.12-2.73)	*	1.87 (1.20-2.91)	**
Dead-end	2.65 (1.69-4.15)	***	2.77 (1.78-4.33)	***	2.76 (1.78-4.28)	***
Precarious	3.39 (2.21-5.19)	***	3.11 (2.03-4.77)	***	2.59 (1.66-4.03)	***
Optimistic precarious	1.81 (1.11-2.96)	*	1.70 (1.05-2.77)	*	1.58 (0.97-2.58)	
Skilled contractor	1.24 (0.61-2.53)		1.44 (0.7-2.95)		1.60 (0.79-3.25)	
Job-to-job	1.84 (1.14-2.97)	*	1.91 (1.18-3.09)	**	1.87 (1.16-3.03)	*
Age (ref. = <30)						
31-50			0.77 (0.62-0.96)	*	0.79 (0.64-0.97)	*
>51			0.69 (0.53-0.89)	**	0.68 (0.53-0.89)	**
Female (ref. = male)			1.25 (1.03-1.51)	*	1.34 (1.11-1.63)	**
Race/ethnicity (ref. = White)						
Black			0.70 (0.53-0.93)	*	0.65 (0.49-0.85)	**
Other			0.97 (0.59-1.59)		1.02 (0.63-1.64)	
Hispanic			1.13 (0.85-1.52)		1.01 (0.74-1.36)	
Nativity (ref. = Born in U.S.)			0.74 (0.52-1.04)		0.74 (0.52-1.05)	
Education (ref. = less than HS)						
High school					0.76 (0.57-1.01)	
Junior college					0.44 (0.27-0.72)	**
Bachelors					0.41 (0.28-0.60)	***
Graduate school					0.37 (0.23-0.62)	***
Intercept	0.07 (0.05-0.1)	***	0.08 (0.06-0.12)	***	0.12 (0.07-0.19)	***
AIC	3584.9		3570.3		3531.5	
Log-likelihood ratio test comparing each model with the previous one			χ^2=28.54, *df*=7, *p*<0.001		χ^2=46.80, *df*=4, *p*<0.001	

Source: Authors' compilation based on General Social Survey (Smith et al. 2013).
Notes: All models are adjusted for survey year. * p-value < 0.05; ** p-value < 0.01; *** p-value < 0.001.

Table A8. Association between employment quality type and occupational injury (incident rate ratios).

	Basic Model		Demographics		Demographics & Education	
	Estimate (95% CI)		Estimate (95% CI)		Estimate (95% CI)	
EQ typology (ref. = SER-like jobs)						
Portfolio	0.72 (0.36-1.43)		0.65 (0.33-1.30)		0.85 (0.42-1.71)	
Inflexible skilled	3.86 (2.22-6.70)	***	3.39 (1.91-6.01)	***	3.61 (2.04-6.39)	***
Dead-end	4.19 (2.35-7.47)	***	4.01 (2.25-7.16)	***	3.93 (2.21-7.00)	***
Precarious	3.06 (1.69-5.52)	***	2.73 (1.49-4.98)	**	2.30 (1.25-4.25)	**
Optimistic precarious	1.1 0(0.52-2.35)		1.05 (0.50-2.22)		0.97 (0.46-2.05)	
Skilled contractor	2.08 (0.96-4.52)		2.04 (0.93-4.46)		2.26 (1.03-4.96)	*
Job-to-job	2.13 (1.06-4.28)	*	2.17 (1.08-4.37)	*	2.12 (1.05-4.25)	*
Age (ref. = <30)						
31-50			0.67 (0.52-0.87)	**	0.68 (0.53-0.88)	**
>51			0.48 (0.34-0.68)	***	0.49 (0.34-0.70)	***
Female (ref. = male)			0.70 (0.55-0.90)	**	0.76 (0.59-0.97)	*
Race/ethnicity (ref. = White)						
Black			0.61 (0.44-0.84)	**	0.55 (0.39-0.76)	***
Other			1.00 (0.5-1.99)		1.10 (0.57-2.15)	
Hispanic			1.03 (0.70-1.51)		0.90 (0.61-1.33)	
Nativity (ref. = Born in U.S.)			0.99 (0.65-1.51)		1.01 (0.66-1.56)	
Education (ref. = less than HS)						
High school					0.75 (0.53-1.04)	
Junior college					0.64 (0.39-1.04)	
Bachelors					0.40 (0.24-0.68)	***
Graduate school					0.28 (0.15-0.51)	***
Intercept	0.1 (0.07-0.15)	***	0.18 (0.11-0.31)	***	0.26 (0.15-0.46)	***
AIC	6893		6760.7		6663.4	
Log-likelihood ratio test comparing each model with the previous one			χ^2=146.3, *df*=7, *p*<0.001		χ^2=105.20, *df*=4, *p*<0.001	

Source: Authors' compilation based on General Social Survey (Smith et al. 2013).
Notes: All models are adjusted for survey year. * p-value < 0.05; ** p-value < 0.01; *** p-value < 0.001.
